# Menopausal Hormone Therapy Is Associated with Having High Blood Pressure in Postmenopausal Women: Observational Cohort Study

**DOI:** 10.1371/journal.pone.0040260

**Published:** 2012-07-11

**Authors:** Christine L. Chiu, Sanja Lujic, Charlene Thornton, Aiden O'Loughlin, Angela Makris, Annemarie Hennessy, Joanne M. Lind

**Affiliations:** 1 School of Medicine, University of Western Sydney, Penrith, New South Wales, Australia; 2 Department of Renal Medicine, Liverpool Hospital, Liverpool, Sydney, Australia; University of Maryland School of Medicine, United States of America

## Abstract

**Background:**

The relationship between menopausal hormone therapy (MHT) and cardiovascular risk remains controversial, with a number of studies advocating the use of MHT in reducing risk of cardiovascular diseases, while others have shown it to increase risk. The aim of this study was to determine the association between menopausal hormone therapy and high blood pressure.

**Methods and Findings:**

A total of 43,405 postmenopausal women were included in the study. Baseline data for these women were sourced from the *45 and Up Study*, Australia, a large scale study of healthy ageing. These women reported being postmenopausal, having an intact uterus, and had not been diagnosed with high blood pressure prior to menopause. Odds ratios for the association between MHT use and having high blood pressure were estimated using logistic regression, stratified by age (<56 years, 56–61 years, 62–70 years and over 71 years) and adjusted for demographic and lifestyle factors. MHT use was associated with higher odds of having high blood pressure: past menopausal hormone therapy use: <56 years (adjusted odds ratio 1.59, 99% confidence interval 1.15 to 2.20); 56–61 years (1.58, 1.31 to 1.90); 62–70 years (1.26, 1.10 to 1.44). Increased duration of hormone use was associated with higher odds of having high blood pressure, with the effect of hormone therapy use diminishing with increasing age.

**Conclusions:**

Menopausal hormone therapy use is associated with significantly higher odds of having high blood pressure, and the odds increase with increased duration of use. High blood pressure should be conveyed as a health risk for people considering MHT use.

## Introduction

Menopausal hormone therapy (MHT) is primarily prescribed for the treatment of perimenopausal symptoms. A common concern among those who manage menopausal women is the risk of unfavourable effects of menopausal hormonal therapy on long-term health, particularly cardiovascular health. Early observational studies have shown a reduced incidence of coronary heart disease in women receiving MHT, which suggested MHT protected against coronary heart disease. [1] Clinical trials later suggested MHT did not reduce the risk of coronary heart disease and was of no benefit to cardiac health. [2] Re-examination of trial data has since shown a possible reduced risk of coronary heart disease after 6 years of treatment. [3] As a result, there is still much confusion over the effect of MHT on cardiac health.

Hypertension, or high blood pressure, is considered a major risk factor for cardiovascular morbidity and mortality in postmenopausal women. A number of environmental and genetic factor are associated with risk of developing high blood pressure, with increasing age the largest contributor. [4] Studies investigating the relationship between MHT and blood pressure have also been largely inconsistent. Baseline data from the observational arm of the WHI found current MHT use was associated with an increased likelihood of hypertension. [5] Various clinical trials in normotensive women have either reported no difference in blood pressure with MHT, [6] a decrease in blood pressure with MHT use, [7] or a slight increase in blood pressure with MHT use, [2] compared with placebo. Similarly, studies in hypertensive postmenopausal women have either shown a slight decrease in blood pressure with MHT use, [8] or no effect on blood pressure with MHT use. [9].

Our study investigated the relationship between the use of MHT and prevalence of high blood pressure, in Australian postmenopausal women aged 45 years and over, the *45 and Up Study*. The aim of this current study was to ascertain the association between MHT use and high blood pressure, and whether the number of years spent taking MHT is associated with having high blood pressure.

## Results

**Figure 1:**
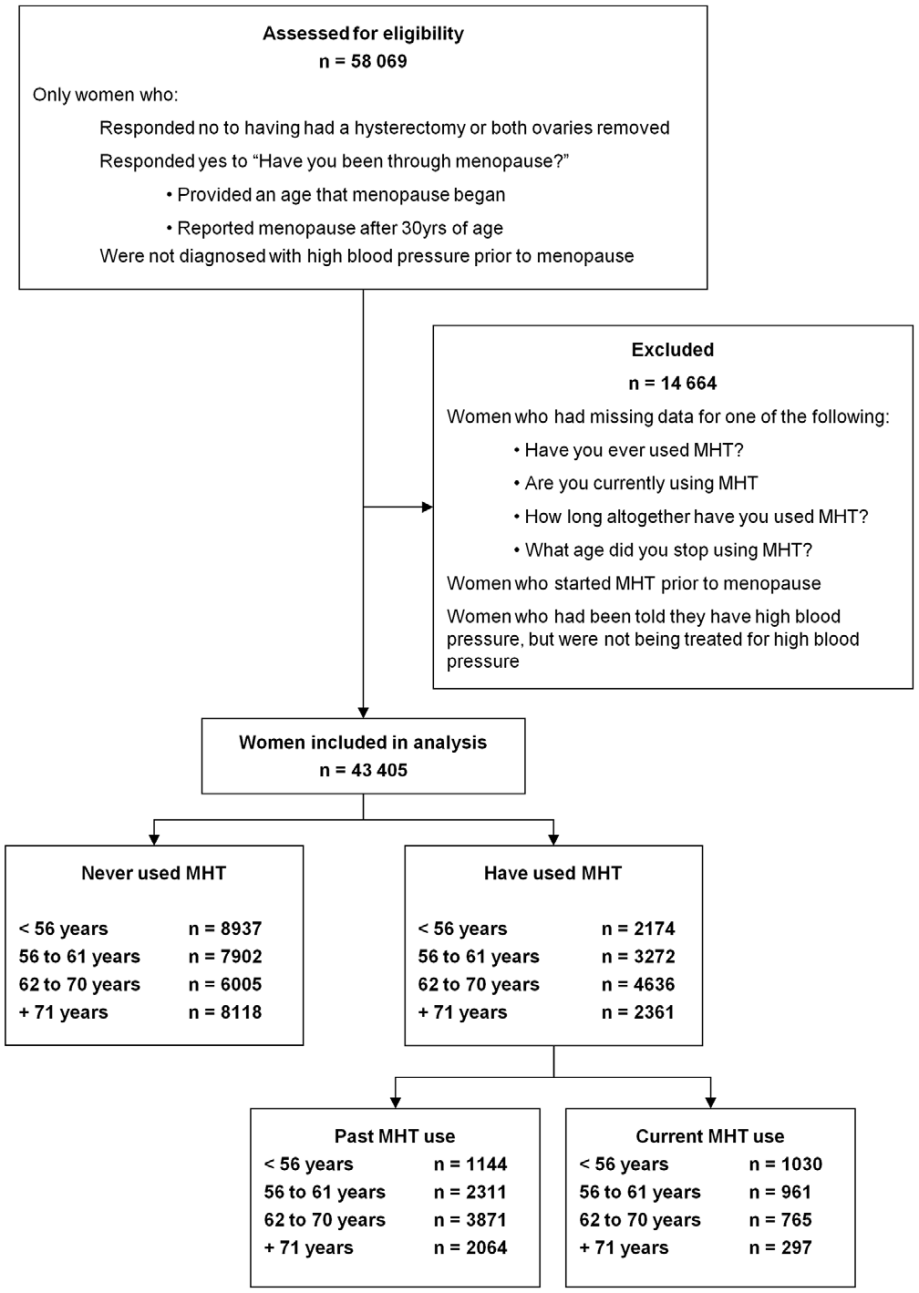
Participants included in the study.

A total of 43,405 women were included in the study (Fig. 1). These women were postmenopausal, had reported to having an intact uterus, had gone through menopause, had not started MHT treatment prior to menopause, and had not been diagnosed with high blood pressure prior to menopause. Of the 12,443 women who had used MHT (past or current), 2,536 (20%) reported having high blood pressure compared to 5,149 (17%) of the 30,962 women who had never used MHT.

Demographic and lifestyle characteristics of MHT users, compared to women who have never used MHT are outlined in Table 1. Women with a family history of high blood pressure were more likely to use MHT than women without a family history of high blood pressure. MHT use was significantly more common in women who reported sufficient levels of physical activity, who were past smokers, who reported drinking alcohol, and who had a history of oral contraceptive use. Women who were obese (BMI greater 30), were current smokers, and had given birth were less likely to use MHT compared with women of a healthy BMI, women who had never smoked, and women who had never given birth, respectively. Country of origin and income were not significantly associated with MHT use.

**Table 1:** Socio-demographic factors and health risk factors associated with MHT use.

Characteristics		N (% column)	% MHT	Odds ratio (99% CI)†
**Country of origin**	Australia	32 360 (75)	29	1.00
	Other	10 680 (25)	28	1.06 (0.99 to 1.13)
**Income**	<$30K	13 319 (30)	28	1.00
	$30K – $70K	10 908 (25)	31	1.10 (1.01 to 1.19)‡
	$70K+	8 460 (19)	28	0.92 (0.84 to 1.00)
	Did not disclose	10 718 (25)	28	1.01 (0.94 to 1.09)
**Family history of HBP**	No	21 882 (50)	28	1.00
	Yes	21 523 (50)	30	1.17 (1.05–1.18)‡
**BMI**	<25	19 905 (46)	29	1.00
	25–30	13 069 (30)	30	1.02 (0.96 to 1.09)
	30+	7 063 (16)	27	0.91 (0.83 to 0.99)‡
**Smoking status**	Never	28 276 (65)	27	1.00
	Past	12 094 (28)	33	1.14 (1.07 to 1.21)‡
	Current	2 840 (7)	27	0.82 (0.73 to 0.93)‡
**Alcohol (drinks/week)**	0	16 994 (39)	25	1.00
	1–5	11 461 (26)	30	1.23 (1.14 to 1.32)‡
	6–10	8 467 (20)	32	1.30 (1.20 to 1.41)‡
	11+	5 517 (13)	34	1.36 (1.24 to 1.49)‡
**Physical activity**	Insufficient	13 028 (30)	26	1.00
	Sufficient	30 377 (70)	30	1.18 (1.11 to 1.26)‡
**Past oral contraceptive use**	No	10 377 (24)	17	1.00
	Yes	32 692 (75)	32	2.73 (2.51 to 2.97)‡
**Have you ever given birth?**	No	5 197 (12)	30	1.00
	Yes	37 954 (87)	29	0.91 (0.83 to 0.99)‡

% MHT – the percentage of women who responded yes to having ever used MHT. Percentages do not consistently total to 100% due to missing values.

† Adjusted for age, country of origin, income level, BMI, smoking status, alcohol consumption, physical activity, family history of high blood pressure, history of oral contraceptive use, age at menopause, and whether a woman had given birth.

‡ p<0.01.

**Figure 2:**
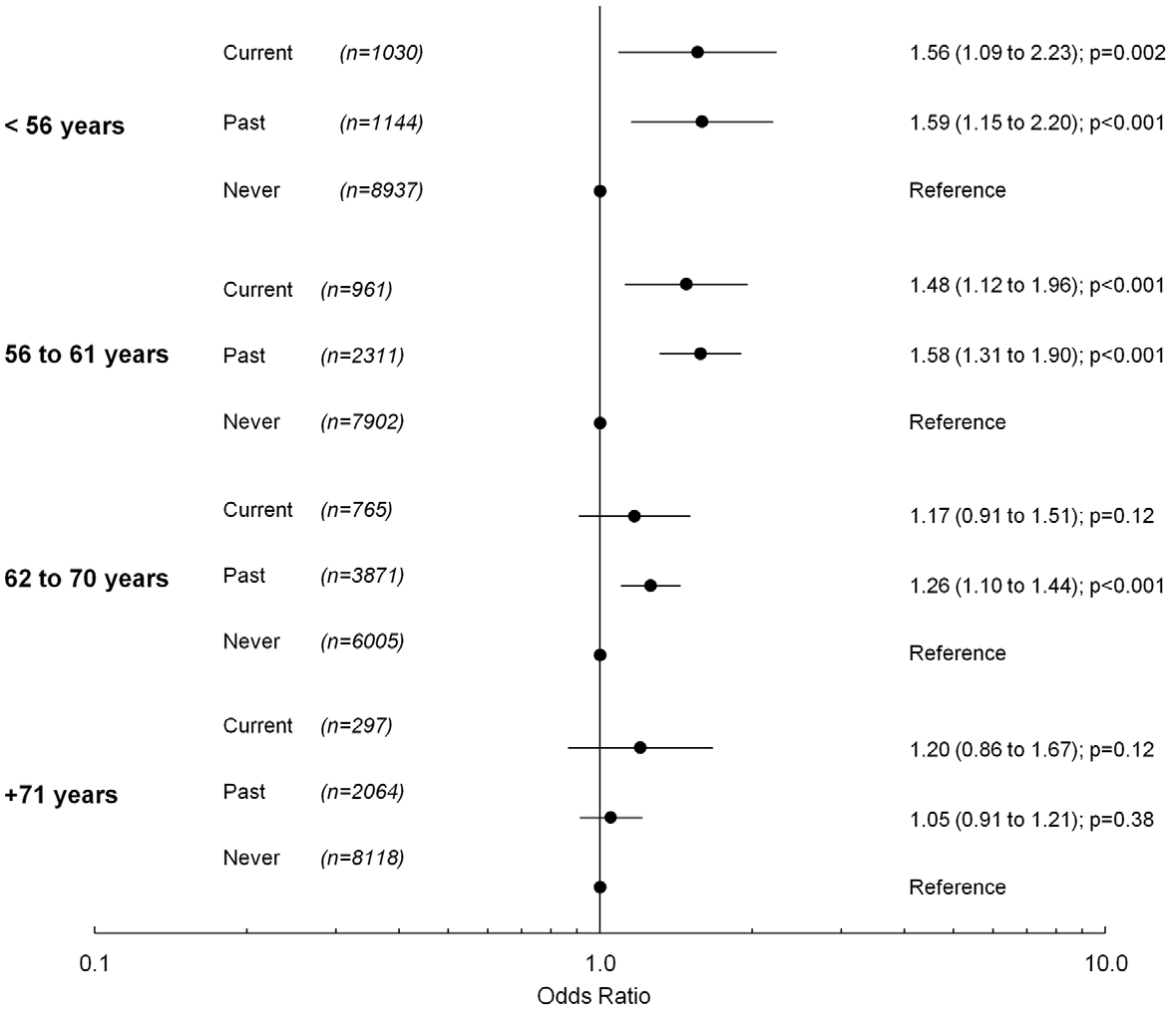
The odds for having high blood pressure in women who have ever used MHT compared with women who have never used MHT, stratified by current age.

The average age for women who had ever used MHT was 63.5 years and 63.7 years for women who had never used MHT (β = 0.14, 99% confidence interval −0.14 to 0.42; p = 0.21). In younger women (<56 years and 56–61 years), analysis looking at current MHT exposure found a significantly higher odds for having high blood pressure in both current and past users of MHT, when compared to women who had never used MHT (Figure 2). In women aged between 62–70 years, there were significantly higher odds of having high blood pressure in past users of MHT compared to women who had never used MHT (Figure 2). In older women (+71 years), there was no association between MHT use and having high blood pressure (Figure 2). There was no significant difference in odds for having high blood pressure between past and current users of MHT in any age group, and as a result, subsequent analysis combined both past and current users of MHT.

The average age at menopause was 48.2 years for women who had ever used MHT and 49.8 years in women who had never used MHT (β = 1.53, 1.41 to 1.66; p<0.001). The average age for when women initiated MHT treatment was 51.6 years. The average age when high blood pressure was first diagnosed was 58.5 years in women who had ever used MHT and 61.3 years in women who had never used MHT (β = 2.8, 2.20 to 3.36; p<0.001).

**Figure 3:**
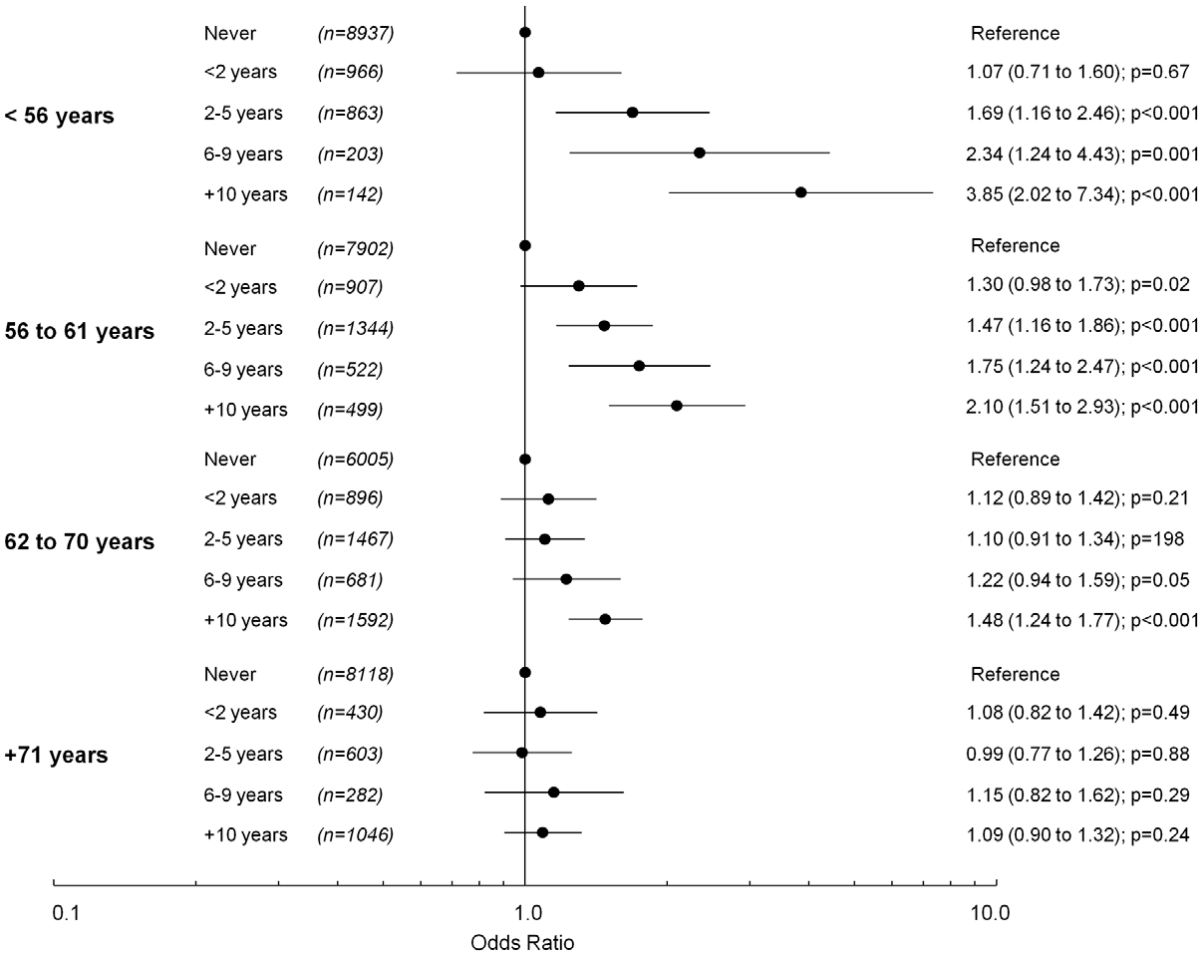
The odds of having high blood pressure depending on duration of MHT use compared to women who have never used MHT, stratified by current age.

Duration of MHT use was associated with an overall increase in odds for having high blood pressure, with women who had used MHT for longer time periods having increased odds for having high blood pressure (Figure 3). However, the effect of duration of MHT use on odds for having high blood pressure was less evident as women got older.

Within a subgroup of MHT users only, no significant association between when MHT was initiated relative to menopause and having high blood pressure was observed at any age group. Additionally, in women who had been treated with MHT in the past, no association between length of time since MHT exposure ceased and having high blood pressure was observed.

## Discussion

This study found that postmenopausal women who use MHT had higher odds of having high blood pressure, and that the longer a woman uses MHT the higher her odds for having high blood pressure, irrespective of both when MHT was initiated relative to menopause and the number of years since stopping treatment. Additionally, women who had ever used MHT were first diagnosed with high blood pressure 2.8 years earlier than women who had never used MHT and as the age of women increased, the association between MHT use and high blood pressure diminished. The association between the duration of MHT use and high blood pressure also diminished with increased age, demonstrating age as a significant predictor for the development of high blood pressure. [10].

### Strengths and Limitations of the study

The key strength of our study was the large sample size, which enabled us to test whether MHT use and duration of MHT use was associated with high blood pressure. It confirms cross-sectional baseline results from the WHI, [5] and extends these findings by showing that both past and current users of MHT have higher odds of having high blood pressure, and that the association between MHT use and high blood pressure is higher in younger, postmenopausal women. Furthermore, this is the first report that the longer a woman uses MHT the higher her odds for having high blood pressure, and is retained irrespective of when MHT was initiated relative to menopause, or the number of years since ceasing MHT treatment.

A limitation of the study was that it analysed cross-sectional baseline data from the 45 and Up Study and, as a result, the casual nature of associations could not be assessed. Additionally, while several known risk factors for high blood pressure were statistically controlled for, factors known to be associated with altering a woman's risk of having a cardiovascular disease, including the type and dosage of MHT, could not be determined. A further limitation is the study's reliance on self-reported data. This method is prone to potential recall bias, which may lead to under-reporting or over-reporting of age at diagnosis, and menopause status. Bias and over-estimation of effects may have potentially been introduced as women who use MHT will have routine health checks and may be more likely to be diagnosed with high blood pressure. However, research has shown that women who use MHT represent a healthier subgroup of the female population, [11] and supports our data, which shows women who use MHT are more likely to be of a healthy weight and be physically active.

### Comparison with other studies

The relationship between MHT use and blood pressure has been inconsistently reported in the literature. Studies in healthy postmenopausal women have found either no treatment related changes in blood pressure, [6] or a lowering of blood pressure. [7] A study looking at hypertensive postmenopausal women, treated with low doses of oral MHT over a 12 month period, also observed a significant decrease in blood pressure in the MHT group. [8] Baseline data from the observational arm of the WHI showed that current HRT use was associated with a 25% increased likelihood of hypertension, after adjusting for known confounders. [5] Increases in systolic blood pressure with MHT use were also observed in the estrogen plus progestin component of the WHI, [2] and the estrogen only component of the WHI. [12] These studies focused on changes in blood pressure while the women were actively receiving MHT treatment. Our results support the conclusions drawn from the WHI observational baseline study and are consistent with the larger clinical trials, which suggest that current MHT use influences blood pressure. Additionally, our data showed that past MHT use is associated with higher odds of having high blood pressure, and these odds remain regardless of the length of time since last exposure.

The timing of initiation of MHT relative to menopause may influence its effect on cardiovascular disease, with secondary analysis of WHI data observing a trend for reduced CHD in younger women who initiated MHT closer to when menopause began. [3] In our study, no association between the when MHT was initiated relative to the onset of menopause was seen. Whether the initiation of MHT prior to the onset of menopause is beneficial to cardiac health, is currently being investigated in the Kronos Study. [13] Our study only included women who had initiated MHT after menopause, as women who initiate MHT prior to menopause are unable to ascertain their menopause status.

Vasomotor symptoms, such as hot flushes, are the primary reason women seek MHT treatment. [14] Women with and without hot flushes have different vascular responses to MHT. [15] This may explain the different results between studies. The severity of hot flushes have been shown to be an independent predictor of endothelial dysfunction, [16] and have been suggested as a marker of cardiovascular risk. [17] It may be women who require MHT to treat vasomotor symptoms are at an increased risk of cardiovascular disease and high blood pressure, irrespective of whether they use MHT. Alternatively, exposure to MHT may further exacerbate existing endothelial damage, which results in high blood pressure even after treatment has stopped. Our data found no significant difference in odds of having high blood pressure between current and past users, with increased odds observed for both groups, compared to women who had never used MHT. Additionally, the number of years since women were last exposed to MHT was not significantly associated with having high blood pressure, that is, the odds of having high blood pressure remains the same, whether you ceased treatment one year ago or more than a decade ago.

### Implications and future research

Women should be prescribed MHT for as short a time as possible and their cardiovascular health, specifically blood pressure, should be closely monitored both during and after therapy. How endothelial dysfunction is associated with menopausal symptoms and the effect of MHT on the vascular system remains to be investigated. A clinical trial of MHT, which includes an extended period of follow up after cessation of treatment, is required to decipher how MHT treatment leads to higher odds of having high blood pressure. Such a trial should initiate MHT close to menopause and only include women who have never used MHT previously.

### Conclusions

Menopausal hormone therapy use is associated with higher odds of having high blood pressure particularly in younger postmenopausal women. Increased duration of menopausal hormone therapy use is also associated with higher odds of having high blood pressure, and this persists regardless of the time since MHT was initiated after menopause, or the length of time since a women stopped using MHT. Our results align with the current Food and Drug Administration recommendations, [18] that MHT treatment be limited to the shortest possible duration consistent with treatment goals. Women who require MHT should be closely monitored as they may represent a population of women with subclinical cardiovascular disease, and blood pressure should be regularly monitored even after treatment ceases. Furthermore, high blood pressure should be conveyed as a health risk for people considering MHT use.

## Methods

Data was obtained from women who were recruited from the *45 and Up Study*. The methods for the *45 and Up Study* have been described elsewhere. [19] Briefly, the *45 and Up Stud*y is a large scale cohort study of healthy ageing that involves 266,848 men and women aged 45 years and over from the general population of New South Wales, Australia. Individuals were sampled from the Medicare Australia database. Study recruitment commenced in 2006 and was completed in 2009. The *45 and Up Study* received ethics approval from the University of NSW Human Ethics Committee. Although derived from the general population, the relatively low response rate means that the cohort is unlikely to be directly representative of the general population. [19] However, exposure-outcome relationships estimated from the *45 and Up Study* data have been shown to be consistent with another large study of the same population, regardless of the underlying response rate or mode of questionnaire administration. [20].

All of the variables used in this study were derived from self reported data obtained from the *45 and Up Study* baseline questionnaire (available at www.45andUp.org.au). Only data from female participants was included in this study. Only women who responded “No” to having had a hysterectomy or both ovaries removed were included. Only women who responded “Yes” to “Have you been through menopause?” and provided an age that menopause began were included. Women who responded “No”, “Not sure (*because hysterectomy, taking HRT, etc*.)” or “My periods have become irregular” were not included in the study. Women were classified as having ever had high blood pressure according to their response to the question “In the last month have you been treated for high blood pressure?”. Only women who did not report having high blood pressure prior to menopause were included. Women who answered “Yes” to “Has a doctor ever told you that you have: high blood pressure – when not pregnant”, but were not being treated for high blood pressure, were excluded. Women who reported menopause before the age of 30 years were excluded. Women who reported current oral contraceptives use were excluded. Variables were classified according to the groups in Table 1.

For ‘Country of birth’, participants were classified according to whether they had been born in Australia or born in a country other than Australia. Physical activity levels were assessed using questions from the Active Australia Survey. Vigorous activity received twice the weighting of moderate or walking activity, [21] and individuals were categorised into sufficient (>150 minutes per week) or insufficient levels. [22].

Regarding use of MHT, women were asked “Have you ever used hormone replacement therapy?” and “If Yes, for how long altogether have you used hormone replacement therapy?” (in years). Women were also asked “Are you currently taking hormone replacement therapy?” and “If No, at what age did you stop?”. MHT use was analysed as a dichotomous variable (ever used MHT/never used MHT), and categorical current MHT exposure (never used MHT, past use, and current use), with women who had never used MHT as the reference group. Duration of MHT use in years was analysed as a categorical variable, divided into quartiles (never; <2 years; 2–4 years; 5–9 years; and 10 or more years), with women who had never used MHT as the reference group. Women with missing data for these variables were excluded.

For women who had used MHT in the past, the age that these women began MHT treatment was calculated by subtracting the number of years that they had used MHT from the age that these women stopped taking MHT. For women currently using MHT, the number of years that they had been using MHT was subtracted from their current age. The age that menopause occurred was then subtracted from the age that MHT use began, to calculate the length of time between when MHT use began relative to menopause. Women who began using MHT prior to menopause were excluded. The length of time since MHT treatment was stopped was calculated for women who had used MHT in the past, by subtracting the woman's current age from the age that MHT was stopped.

Odds ratios and 99% confidence intervals were estimated using logistic regression. Both crude and adjusted odds ratios were calculated and descriptions refer to adjusted odds ratios unless otherwise specified. Odds ratios were adjusted for demographic and lifestyle factors using the categories in Table 1, with an additional category for missing values. Tests for interactions between covariates and MHT use, with odds for high blood pressure were performed. A significant interaction between age and MHT use with having high blood pressure was observed. As a result, women were stratified according to age and divided into quartiles (<56 years, 56–61 years, 62–70 years and 71 years or older). Odds ratios were calculated for each age group. No other significant interactions were observed. All statistical tests were two-sided, using a significance level of p<0.01 to partially account for multiple testing issues. [3], [23], [24] Conclusions were drawn based on both significance and the effect size.
